# Angle change of the A-domain in a single SERCA1a molecule detected by defocused orientation imaging

**DOI:** 10.1038/s41598-021-92986-3

**Published:** 2021-07-01

**Authors:** Takanobu A. Katoh, Takashi Daiho, Kazuo Yamasaki, Stefania Danko, Shoko Fujimura, Hiroshi Suzuki

**Affiliations:** 1 Department of Physics, Faculty of Science, Gakushuin University, Toshima-ku, Tokyo 171-8588 Japan; 2 Department of Biochemistry, Asahikawa Medical University, Midorigaoka-higashi, Asahikawa, 078-8510 Japan; 3 Present Address: Laboratory for Organismal Patterning, RIKEN Center for Biosystems Dynamics Research, RIKEN, Minatojima-minamimachi, Chuo-ku, Kobe, Hyogo 650-0047 Japan; 4 Present Address: AIST-UTokyo Advanced Operando-Measurement Technology Open Innovation Laboratory (OPERANDO-OIL), National Institute of Advanced Industrial Science and Technology (AIST), Kashiwa, 277-8565 Japan

**Keywords:** Biophysical chemistry, Single-molecule biophysics, Membrane proteins, Ion transport

## Abstract

The sarcoendoplasmic reticulum Ca^2+^-ATPase (SERCA) transports Ca^2+^ ions across the membrane coupled with ATP hydrolysis. Crystal structures of ligand-stabilized molecules indicate that the movement of actuator (A) domain plays a crucial role in Ca^2+^ translocation. However, the actual structural movements during the transitions between intermediates remain uncertain, in particular, the structure of *E*2PCa_2_ has not been solved. Here, the angle of the A-domain was measured by defocused orientation imaging using isotropic total internal reflection fluorescence microscopy. A single SERCA1a molecule, labeled with fluorophore ReAsH on the A-domain in fixed orientation, was embedded in a nanodisc, and stabilized on Ni–NTA glass. Activation with ATP and Ca^2+^ caused angle changes of the fluorophore and therefore the A-domain, motions lost by inhibitor, thapsigargin. Our high-speed set-up captured the motion during *E*P isomerization, and suggests that the A-domain rapidly rotates back and forth from an *E*1PCa_2_ position to a position close to the *E*2P state. This is the first report of the detection in the movement of the A-domain as an angle change. Our method provides a powerful tool to investigate the conformational change of a membrane protein in real-time.

## Introduction

**Figure 1:**
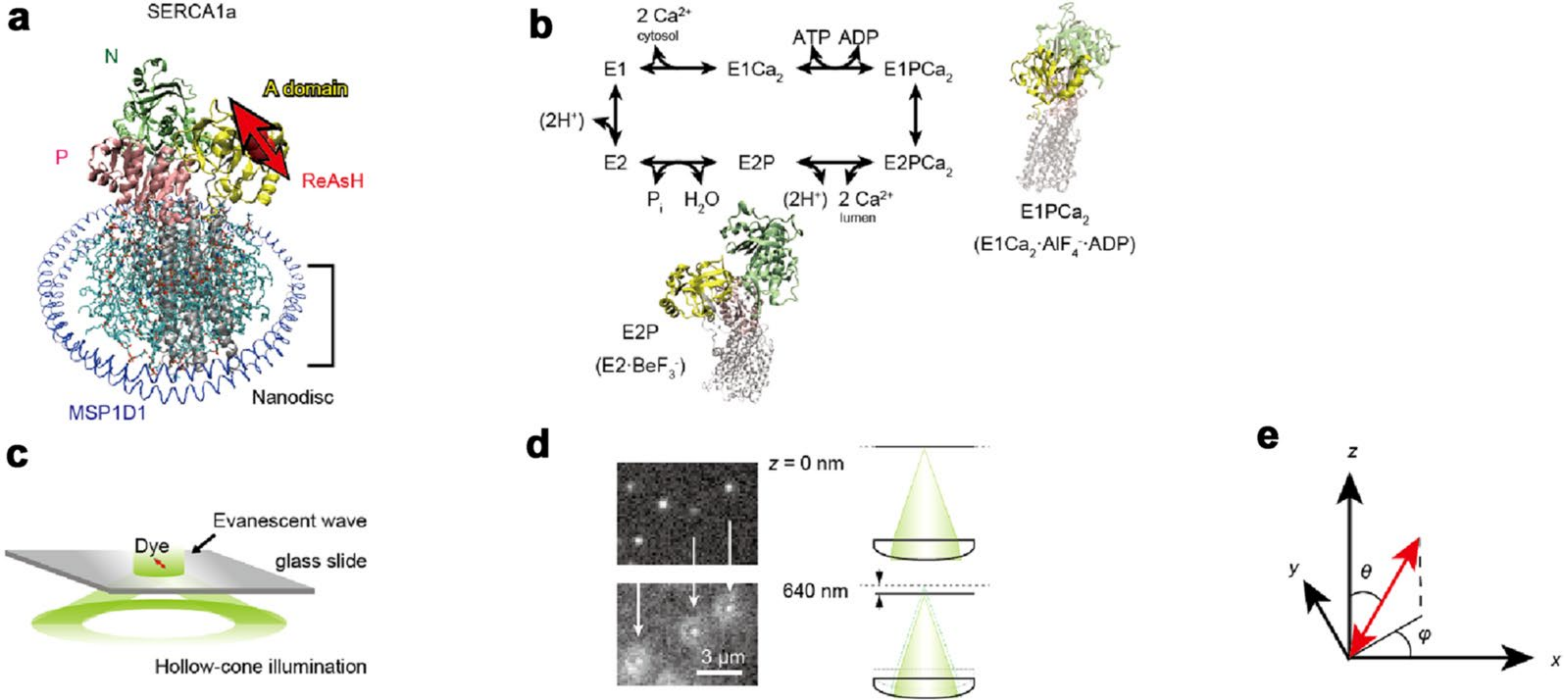
Schematic of SERCA1a molecule and defocused orientation imaging. (**a**) A schematic of the experimental setup. A single SERCA1a molecule was embedded in the nanodisc. Yellow, green, and pink domains represent A, N, P-domains, respectively. Fluorophore ReAsH is attached to its A-domain. Blue structure represents the membrane scaffold protein of the nanodisc (MSP1D1). Double headed red arrow represents presumed dipole moment of ReAsH. (**b**) Reaction scheme of the Ca^2+^-ATPase and the crystal structures in the *E*1PCa_2_ (*E*1Ca_2_·AlF_4_^−^·ADP) and *E*2P (*E*2·BeF_3_^−^) states (PDB ID code: 1T5T and 2ZBE; Two molecules were aligned using M7-M10 helices). A-domain undergoes a ~ 90° rotation during *E*P isomerization. (**c**) A schematic of the isotropic total internal reflection fluorescence microscopy (iTIRF). The laser beam comes from all directions as a hollow-cone of illumination. The evanescent field produced by the iTIRF contains all polarization components along *x*-, *y*-, and *z*-directions, thus single fluorophores are efficiently excited even though each fluorophore orients in a different direction. (**d**) Image and schematic of the defocused imaging. The objective lens was typically placed 640 nm away from the best focal plane. (**e**) Definition of the zenith (*θ*) and azimuth (*φ*) angle. The *z*-axis represents the optical axis.

Conformational changes in sarcoendoplasmic reticulum Ca^2+^-ATPase (SERCA) lead to the transport of cytosolic Ca^2+^ ions into the sarcoplasmic reticulum against a > 10^4^ concentration gradient^1–4^. SERCA1a is a P-type ion transporting ATPase which is involved in the control of skeletal muscle contraction^5^, and is constructed of three cytosolic domains (a nucleotide-binding (N) domain, a phosphorylation (P) domain, and an actuator (A) domain) and a membranous region (helices M1-M10)^4,6^ (see Fig. 1a). The A-domain, connected with helices M1–M3 via A-M1, A-M2, and A-M3 linkers, plays a crucial role in the Ca^2+^ translocation, ATPase reaction and their coupling^7–13^. The catalytic cycle of Ca^2+^ translocation involves auto-phosphorylation and dephosphorylation of a catalytic residue (Asp351) and proceeds through six intermediates (*E*1, *E*1Ca_2_, *E*1PCa_2_, *E*2PCa_2_ *E*2P, and *E*2, in which *E*1PCa_2_ and *E*2P refer to the phosphorylated intermediate reactive to ADP regenerating ATP and with two occluded Ca^2+^ at transport sites (*E*1PCa_2_), and the one insensitive to ADP and after Ca^2+^ release (*E*2P), respectively; see reaction scheme of Fig. 1b). The A-domain appears to show significant changes in angle during the cycle^1,6,14^. This conformational change in the A-domain is coupled with an alteration of Ca^2+^ affinity inside the membranous region and the opening and closing of a release-gate through the three linkers.

The binding of two cytosolic Ca^2+^ ions into the transport-sites in the transmembrane region forms the *E*1Ca_2_ state through the conformational change of the A-domain^1,15,16^. During the *E*2 to *E*1Ca_2_ transition, the A-domain rotates ~ 110° around an axis approximately perpendicular to the membrane. In the *E*1Ca_2_ state, the cytoplasmic domains show an open structure^6^. During the formation of the *E*1PCa_2_ intermediate, ATP binds and crosslinks the P- and N-domains, and the A-domain undergoes a ~ 30° upward inclination motion. Subsequently, the molecule changes from an ADP-sensitive phosphoenzyme (*E*1P) to an ADP-insensitive one (*E*2P) with a ~ 90° inverse-rotation of the A-domain—the so-called *E*P (phosphoenzyme) isomerization step^17^ (Fig. 1b). After isomerization, Ca^2+^ ions are released to the luminal side^1,17^. However, these findings are derived from static crystal structures and the actual domain movements between conformations of intermediates remain conjectural. In particular, the actual A-domain motion during *E*P isomerization can only be guessed at, due, in part, to the lack of an *E*2PCa_2_ structure, a transient state during *E*P isomerization. Although a change in the distance between the A- and P-domains has been reported^18^, in order to track the detailed dynamics of Ca^2+^ translocation, the angular changes of domains in 3-D space need to be investigated with a high temporal resolution.

In this study, the angular change of the A-domain in a single SERCA1a molecule was measured by defocused orientation imaging. Localization within the spherical coordinate of a single dipole enables us to derive a defocused image that was taken after the displacement the objective typically less than one micron from the in-focus plane of the sample (see Fig. 1c–e). By matching patterns that emerge from the rigorous formation model with 3-D steerable filters to an acquired image^19–21^, both zenith angle (*θ*) and azimuth angle (*φ*) are simultaneously estimated from a single defocused profile. We were able to detect a change in the *θ* and *φ* angles of the A-domain in the same single molecule between *E*1Ca_2_ and a state analogous to *E*2P (*E*2·BeF_3_^−^: *E*2P ground state analog^22^) by stabilizing the molecule sequentially with two different ligands. Furthermore, we observed turnover-dependent motion of the A-domain in real-time. Especially noteworthy, our high-speed set-up was capable of observing the back and forth motion of the A-domain during *E*P isomerization.

## Results

### Preparation of fluorophore-labeled SERCA1a for defocused orientation imaging

**Figure 2:**
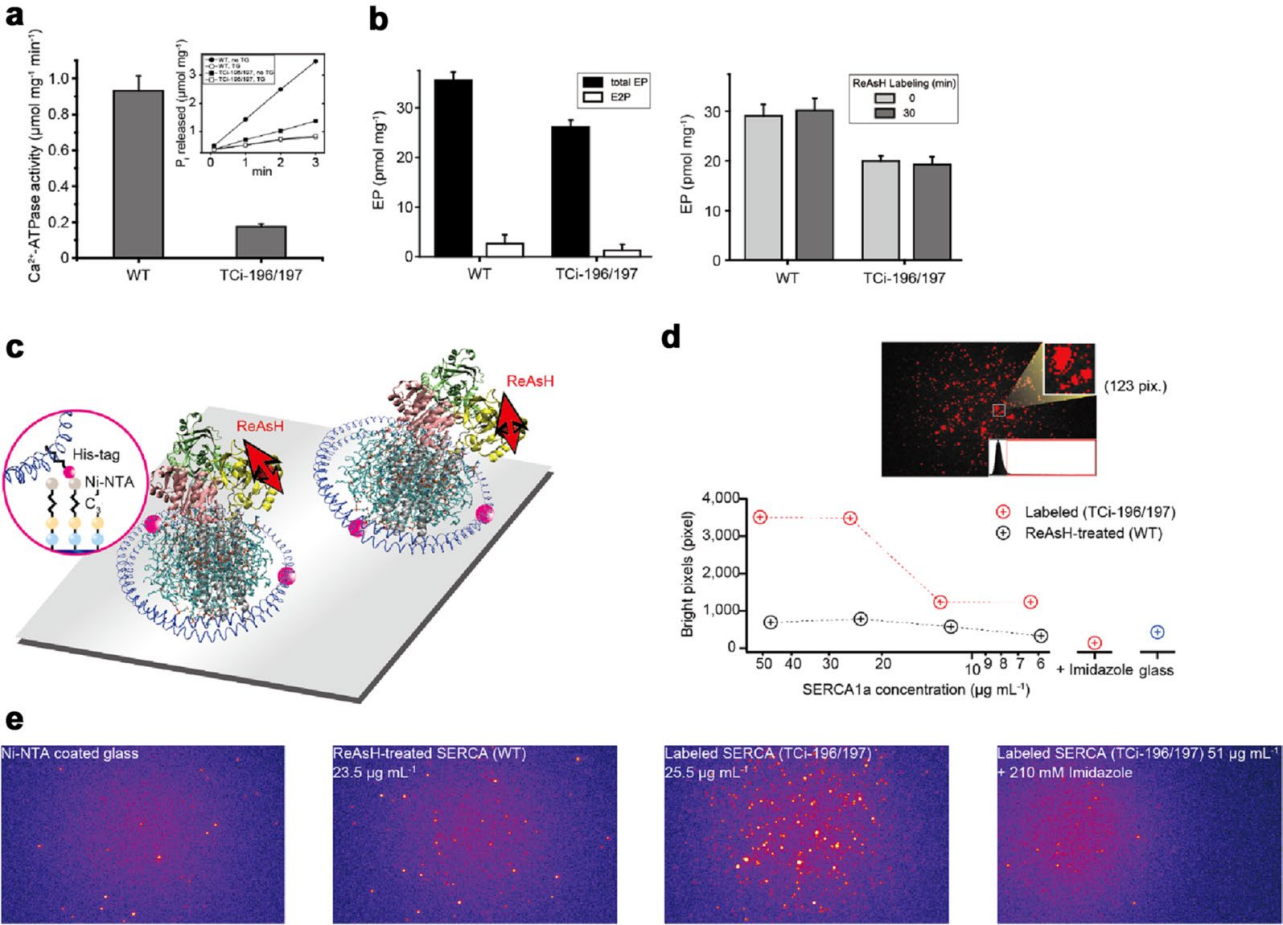
Ca^2+^-ATPase activity, amount of *E*P, and observation of ReAsH attached to the A-domain in SERCA1a molecule. (**a**) Ca^2+^-ATPase activity of expressed SERCA1a obtained by subtracting the ATPase activity determined in the presence of 1 μM TG as described under “Methods” section. The values presented are the mean ± SD (n = 3–4). Typical time courses of P_i_ liberation in the wild type and TCi-196/197 mutant in the absence (*closed symbols*) and presence of TG (*open symbols*) are shown in the *inset*. (**b**) Total *E*P at steady-state and *E*2P fraction. Microsomes expressing wild type or TCi-196/197 mutant were phosphorylated and *E*P was determined as described under “Methods” section (*Left*). There was no impairment of *E*P formation by ReAsH labeling (*Right*). Microsomes expressing wild type or TCi-196/197 mutant were labeled with ReAsH and *E*P was determined as described under “Methods” section. The values presented are the mean ± SD (n = 3–4). (**c**) ReAsH-labeled single SERCA1a molecule was stabilized on Ni–NTA coated glass surface via two his-tags incorporated into the membrane scaffold protein of the nanodisc. The angle of the nanodisc is not controlled because it has only two His-tags, not a three-point attachment. (**d**) SERCA1a concentration dependence of the number of observed bright pixels. To quantify the bright areas, mainly attributed to ReAsH, we calculated the area of bright pixels above the threshold value (*inset*). The number of bright pixels clearly depends on the concentration of the ReAsH-labeled SERCA1a embedded nanodisc (cf. Supplementary Fig. S2). (**e**) Fluorescent images of the glass surface (in the absence of protein), of the ReAsH-treated non-TC-tagged SERCA1a (WT), of the ReAsH-labeled SERCA1a (TCi-196/197) and of the imidazole-treated ReAsH-labeled SERCA1a (TCi-196/197). Red dots represent fluorophores. Note that almost all SERCA1a embedded nanodiscs were detached with 210 mM imidazole.

We expressed a rabbit SERCA1a that possessed an inserted tetra-cysteine (TC) mutation between residues 196 and 197 in the A-domain^8^. Analysis of crystal structures of this region predicts a large angle change towards the P-domain between the *E*1PCa_2_ state and the *E*2P state (Supplementary Fig. S1a). The TC motif was labeled with biarsenical reagent ReAsH (Fig. 1a)^23^. The TC-inserted SERCA1a (TCi-196/197) showed slow Ca^2+^-ATPase activity (Fig. 2a), at ~ 20% of wild type. The ReAsH-labeled species formed *E*P in amounts comparable with that of wild type (Fig. 2b), due to slow *E*P isomerization, deduced from the reduced ATPase activity. Therefore, the TC insertion and ReAsH labeling, while exhibiting restrained isomerization, still permit the normal conformational changes of SERCA1a. Single ReAsH-attached SERCA1a molecules were embedded in single nanodiscs of POPC (1-palmitoyl-2-oleoyl-glycero phosphatidylcholine)^24^, and stabilized on the surface of Ni–NTA coated glass via two his-tags located in the nanodisc (Figs. 1a, 2c). Importantly, fluorescent images showed that the number of bright spots decreases as the concentration of labeled SERCA1a is reduced and the bright spots completely disappear (protein molecules detach) from the Ni–NTA glass with imidazole treatment (Fig. 2d,e and Supplementary Fig. S2), indicating that we are able to detect a signal from the ReAsH probe attached to the nanodisc-embedded SERCA1a.

To observe the defocused image of the ReAsH attached to a single SERCA1a molecule, we constructed an isotropic total internal reflection fluorescence microscope (iTIRF)^21,25^. The evanescent field produced by the iTIRF contains all polarization components along *x*-, *y*-, and *z*-directions, thus single fluorophores are efficiently excited even if each fluorophore oriented in a different direction (Fig. 1c). To obtain the defocused image^19–21^, the objective lens was typically displaced 640 nm away from the best focal plane (Fig. 1d). Precise and stable displacements of the objective were achieved through the use of the perfect-focus system equipped with a Nikon TE2000E inverted microscope, and the relationship between the adjustment and the amount of displacement was independently calibrated by a combination of a 3D tracking method and a piezo electric stage (see “Methods” section). The change in the pattern of the defocused image describes the change in the zenith angle (*θ*) of the axis of the fluorophore, and the orientation of the fan-shaped pattern indicates the azimuth angle (*φ*) of that axis (for a definition of *θ* and *φ* see Fig. 1e). The *θ* and *φ* angles were derived from the matching algorithm for the defocused image^20^. Accuracy of orientation estimation of this algorithm is ~ 5° according to Ref^20^ (see Supplementary Fig. S3).

### Angle change in the A-domain of ligand-stabilized SERCA1a

**Figure 3:**
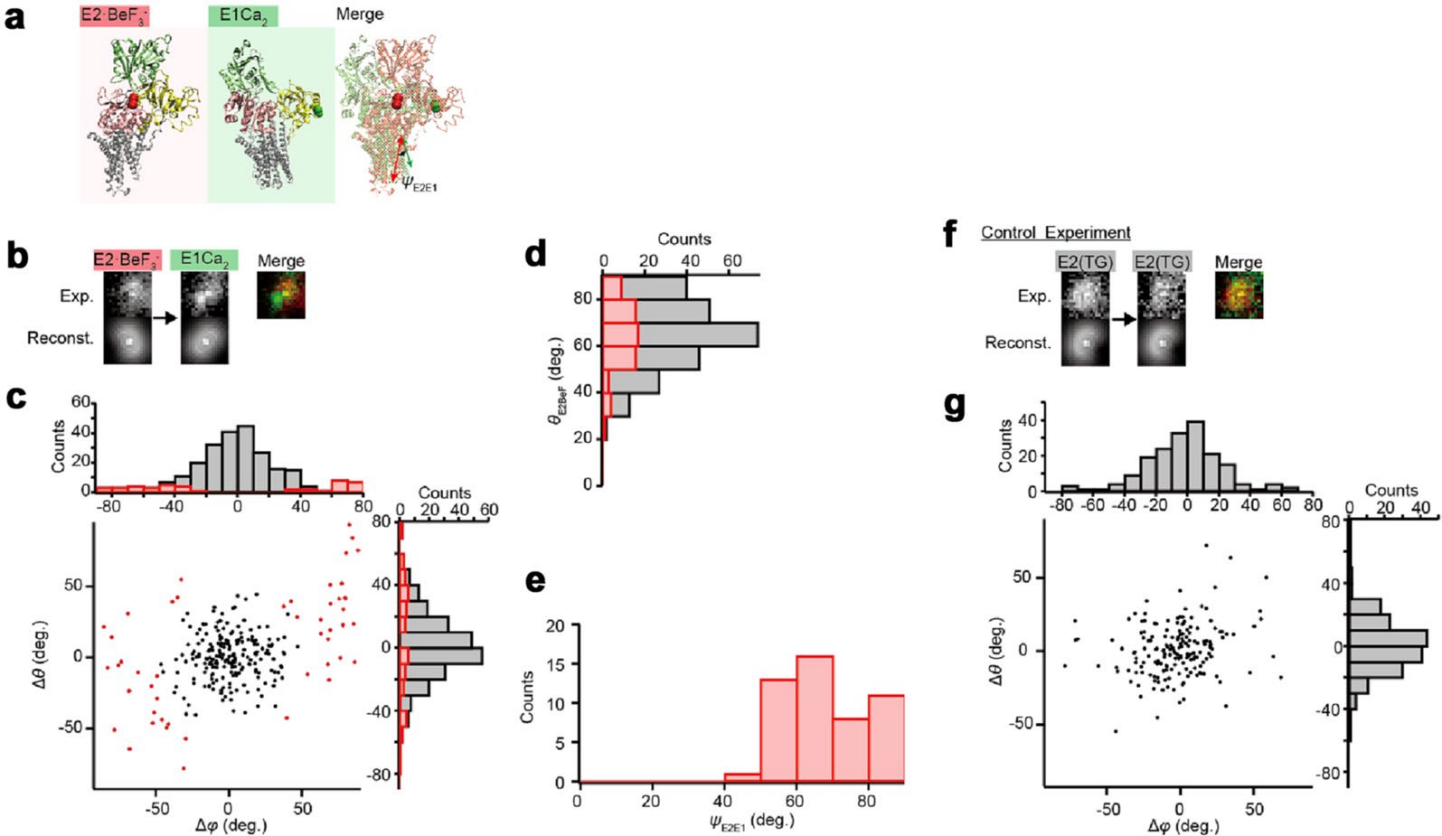
Measurement of the angle change in the A-domain of ligand stabilized single SERCA1a. (**a**) The crystal structures in the *E*2P (*E*2·BeF_3_^−^) and *E*1Ca_2_ states (PDB ID code: 3B9B and 1SU4; Two molecules were aligned using M7-M10 helices). We defined the *ψ*_E2E1_ angle as the angle change of a fluorophore from *E*1Ca_2_ state to *E*2P analogous state (*E*2·BeF_3_^−^) in the same molecule. (**b**) Experimental and reconstructed images in the *E*2P analogous state (*E*2·BeF_3_^−^) and the *E*1Ca_2_ state of the same fluorophore-attached SERCA1a molecule. The estimated (*θ*, *φ*) angles of *E*2P analogous state (*E*2·BeF_3_^−^) and *E*1Ca_2_ state are (106.8°, 202.1°) and (80.0°, 139.3°), respectively. Therefore, the calculated values of Δ*θ* and Δ*φ* are 26.8° and 62.7° in this image. Reconstructed image represents the diffraction pattern rendered from the estimated angle. (**c**) Changes in the *θ* and *φ* angle during the *E*2P analogous state (*E*2·BeF_3_^−^) state transition to the *E*1Ca_2_ state. Red dots represent molecules that show a significant change in angle compared to the control. (**d**) Histogram of *θ* angle in the *E*2P analogous state (*E*2·BeF_3_^−^). Red bars indicate molecules showing a significant change in angle. (**e**) Histogram of *ψ*_E2E1_ angle. The *ψ*_E2E1_ angle was calculated as 67.3° ± 12.3° (*n* = 49 molecules; e.g., *ψ*_E2E1_ in the molecule shown in (**b**) was 67.6°). (**f**) Control: experimental and reconstructed images of TG-bound SERCA1a before (left image) and after (right image) TG solution exchange. The estimated angles of the left image and right image are (*θ*, *φ*) = (61.9°, 154.7°) and (59.7°, 155.7°), respectively. Therefore, the calculated values of Δ*θ* and Δ*φ* are 2.2° and 1.0° in this image and most fluorophores similarly show a small change in angle under these conditions. (**g**) Changes in the *θ* and *φ* angle after TG solution exchange.

**Figure 4:**
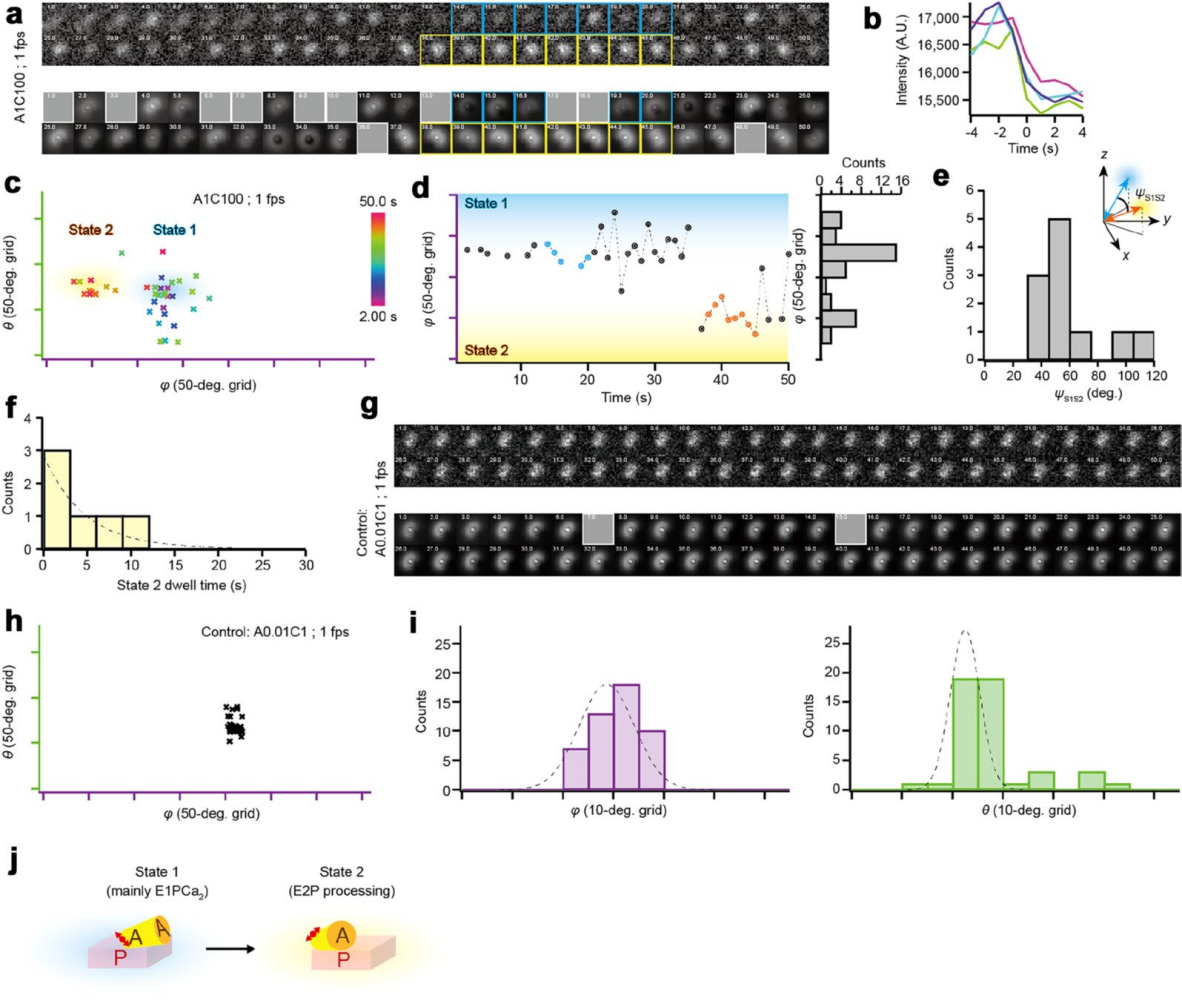
Measurement of the ATP-dependent angle change of the SERCA1a A-domain. (**a**) Sequential defocused images of a fluorophore at 1 µM ATP and 100 µM Ca^2+^ (A1C100; cf. Supplementary Video 1). Time scale described in upper left of each image is in seconds. (*Upper panel*) Experimental images were taken at 1 s intervals. Approximately half of the molecules showed two dwell states (cyan and yellow frames correspond *cyan* and *orange points* in (**d**), respectively). (*Lower panel*) Reconstructed images represent the diffraction pattern rendered using the angles derived from the matching algorithm. Frames covered with gray square are eliminated frames where the matching algorithm failed to estimate angles or estimated incorrect values. (**b**) Typical intensity change of four molecules showing a single bleaching step. (**c**) The *θ* and *φ* angles of the fluorophore at 1 µM ATP and 100 µM Ca^2+^. The color of each point represents the time, as shown in the color bar. Note that the *θ* and *φ* angles are relative because the orientation of the SERCA1a molecule is not controlled in our system. (**d**) Time course of the *φ* angle of the fluorophore at 1 µM ATP and 100 µM Ca^2+^. (*Right*) Histogram of the *φ* angle. The two populations represent the states 1 and 2. (**e**) Histogram of *ψ*_S1S2_ angle between the two dwell states [e.g., *ψ*_S1S2_ angle is calculated as 58.8° using *cyan* and *orange points* in (**d**)]. The calculated *ψ*_S1S2_ angle is 59.5° ± 24.3° (*n* = 11 molecules). Inset schematically shows the definition of the *ψ*_S1S2_ angle. (**f**) A histogram of the dwell time of State 2 (*n* = 6 dwells from 6 molecules). Dotted line indicates exponential fits of which the rate constant was ~ 0.2 s^−1^. (**g**) Control: Sequential defocused images of a fluorophore at 0.01 µM ATP and 1 µM Ca^2+^ taken at 1 s intervals (A0.01C1). In this condition, almost all the molecules are in the *E*1Ca_2_ state, but due to the suboptimal ATP concentration cannot form *E*P. Not a single molecule showed a change in angles. (**h**) The *θ* and *φ* angles of the fluorophore in (**g**). (**i**) A histogram of the *φ* and *θ* angle. The dotted line indicates the Gaussian fit, where *σ*_φ_ = 5.5°and *σ*_θ_ = 2.9°. (**j**) Schematic of the motion of the A-domain, based on the observation in this figure (**a**–**f**). This large azimuthal rotation of fluorophore most likely represents the large azimuthal rotation of the A-domain (see “Discussion” section).

To demonstrate that the A-domain has the ability to change angle while the nanodisc is stabilized on the glass surface, we measured the change in the angle between two different states using the same ReAsH-labeled SERCA1a molecule. *E*2·BeF_3_^−^, a stable analog for the ADP-insensitive *E*P of SERCA1a^22^, is decomposed to *E*1Ca_2_ by high millimolar Ca^2+^ (cf. Figs. 1b, 3a). We compared the defocused image of ReAsH attached to the *E*2P analogous state (*E*2·BeF_3_^−^) of SERCA1a (*Left panel* of Fig. 3b) to that of the same protein molecule after infusion of a solution containing 20 mM Ca^2+^ (*E*1Ca_2_ state; *Right panel* of Fig. 3b) and plotted the changes in angles in Fig. 3c. Note that we selected molecules which show the same intensity as that of a molecule bleached in a single step (cf. Fig. 4b) and which keep the same *xy*-position after infusion. This ensures that each fluorescence is derived from the same molecule and that it is a single molecule^26^. In the *E*2P analogous state (*E*2·BeF_3_^−^), the *θ* angle shows a single population of 65.4° ± 14.3° (*n* = 49; Red Bars in Fig. 3d), suggesting that these SERCA1a-nanodiscs are likely bound to the glass at a particular angle (see “Discussion” section).

We selected only the molecules that could transform from the *E*2P analogous state (*E*2·BeF_3_^−^) to the *E*1Ca_2_ state and ignored both the molecules unable to undergo the transition and the bright spots derived intrinsically from the glass surface (cf. bright spots in the image of Ni–NTA coated glass in Fig. 2e). To determine the angle distribution of a stabilized control molecule, thapsigargin (TG), a highly specific and subnanomolar affinity SERCA inhibitor, was perfused over the ReAsH labeled SERCA1a, which brings it to an extremely stable *E*2 (TG), a form analogous to the *E*2 state^27,28^. The fluorophores then showed random changes in angles before and after the solution exchange (*n* = 180; Fig. 3f,g). The width of this distribution, *σ* = 11.8°, was estimated by fitting to the derived histogram (Fig. 3g). Even though the molecules undergo perfusion, this narrow distribution indicates the stability of the angle of the fluorophore, and therefore the stability in the angle of the SERCA1a molecules and nanodiscs, ensuring that detection of a larger angle change must reflect angular changes in the A-domain associated with the SERCA1a conformational changes.

To select molecules that transform from the *E*2P analogous state (*E*2·BeF_3_^−^) to the *E*1Ca_2_ state, we defined that those molecules showing a significant change in angle, i.e., > 3*σ* of 35.4°, have undergone the conformational change. We found that 19.4% of molecules showed a significant change in angle between the *E*2P analogous state (*E*2·BeF_3_^−^) and the *E*1Ca_2_ state when compared to the TG bound molecules (*n* = 253; Fig. 3b,c) and used these for analysis. Here, we defined the *ψ* angle as the angle change derived from an inner product of two angles (see Fig. 3a). The *ψ*_E2E1_ angle between *E*2P (*E*2·BeF_3_^−^) and *E*1Ca_2_ was determined as 67.3° ± 12.3° (*n* = 49 molecules; red in Fig. 3c,e). This angle change is in excellent agreement with the expected value of 50°–60°, estimated from the crystal structures (PDB ID code: 3B9B for *E*2·BeF_3_^−^ and 1SU4, 2C9M, 3J7T, and 5XA7 for *E*1Ca_2_; see “Methods” section).

### Angle change of A-domain in the presence of Ca^2+^ and ATP

To detect the motion accompanying the overall enzyme reaction, we observed sequential defocused images of a ReAsH attached to SERCA1a in the presence of Ca^2+^ and ATP with a time resolution of 1 s (1 frame per second). To decrease the ATP-dependent turnover rate of SERCA1a, we performed the experiments at 5 °C. Because of the rate of *E*P decay (the rate limiting step of the ATPase cycle) of ~ 0.06 s^−1^ in the wild type (cf. Ref^29^) and taking into consideration the reduced ATPase activity in the TCi-196/197 mutant (Fig. 2a), we were able using these conditions and set-up to capture almost the whole ATP-turnover cycle. Although a large proportion of bright spots did not show a change in angles, ~ 1% of the ReAsH-labeled SERCA1a did at 1 µM ATP and 100 µM Ca^2+^ (Fig. 4a and Supplementary Video 1). Note that reconstructed images in Fig. 4a represent the diffraction pattern rendered using the angle estimated from experimental images. This motion was completely absent when the *E*1Ca_2_ state was formed at subsaturating ATP concentration (0.01 µM, cf. the reported *K*_d_ for MgATP binding to SERCA1a is 6.1 µM^30^) and in the molecules stabilized by TG, confirming that this angle change is driven by Ca^2+^ and ATP (Fig. 4g–i, Supplementary Fig. S4). We selected the molecules that show obvious changes in angles with time and obtained the trajectory of *θ* and *φ* angles by analyzing the defocused images. Note again that these selected molecules show a single bleaching step or have the same intensity of a molecule that exhibits a single bleaching step (Fig. 4b), ensuring that each fluorescence is derived from a single molecule^26^. The trajectory of these molecules showed ~ 2 dwell states, separated along the *φ* axis (light cyan and light orange in Fig. 4c). The time course of the *φ* angle change and its histogram clearly indicate two distinct dwell states (Fig. 4d). Considering the major accumulation of ADP-sensitive *E*P at steady-state (*E*1P; compare the amount of total *E*P and *E*2P in Fig. 2b), we can assume that most molecules dwell in the *E*1PCa_2_ state. Therefore, the longest dwell, termed State 1 (light cyan in Fig. 4c,d), comprises molecules mainly at *E*1PCa_2_ (cf. Fig. 4j). Because of the reaction cycle rate constants of SERCA1a and the expected change in angle derived from crystal structures (Supplementary Fig. S1b)^29^, the other dwell, termed State 2 (light orange in Fig. 4c,d; cf. Fig. 4j), should occur during *E*2P processing, i.e., *E*2PCa_2_ → *E*2P → *E*2 (cf. Fig. 1b). The dwell time of State 2 was fitted by an exponential function of which the rate constant was ~ 0.2 s^−1^ (*n* = 6 molecules; Fig. 4f). This rate is comparable to the postulated rate of the *E*2P processing (see “Discussion” section). To compare the angle with the crystal structure, we calculated the change in angles between State 1 and State 2 as an inner product of *ψ*_S1S2_ using the *θ* and *φ* angles. The molecules forming the *E*1Ca_2_ state show a stable angle with a narrow distribution of *σ* ~ 5° (Fig. 4h,i; (*σ*_θ_, *σ*_φ_) = (2.9°, 5.5°)). Therefore, we defined the stable point as the frame in which the fluorophore remains at the same angle within ~ 3*σ* of 15° in each frame. The *ψ*_S1S2_ angle of 59.5° ± 24.3° (*n* = 11 molecules; Fig. 4e) was calculated using the points that show stable angles in *θ* and *φ* over 3 s (cyan and orange points in Fig. 4d). This is comparable to the estimated angle of ~ 60°, derived from the crystal structures of *E*1Ca_2_·AlF_4_^−^·ADP (an *E*1PCa_2_ model, transition state analog of *E*1PCa_2_ formation by ATP) and *E*2·BeF_3_^−^ (PDB ID code: 1T5T and 2ZBE; see “Methods” section).

**Figure 5:**
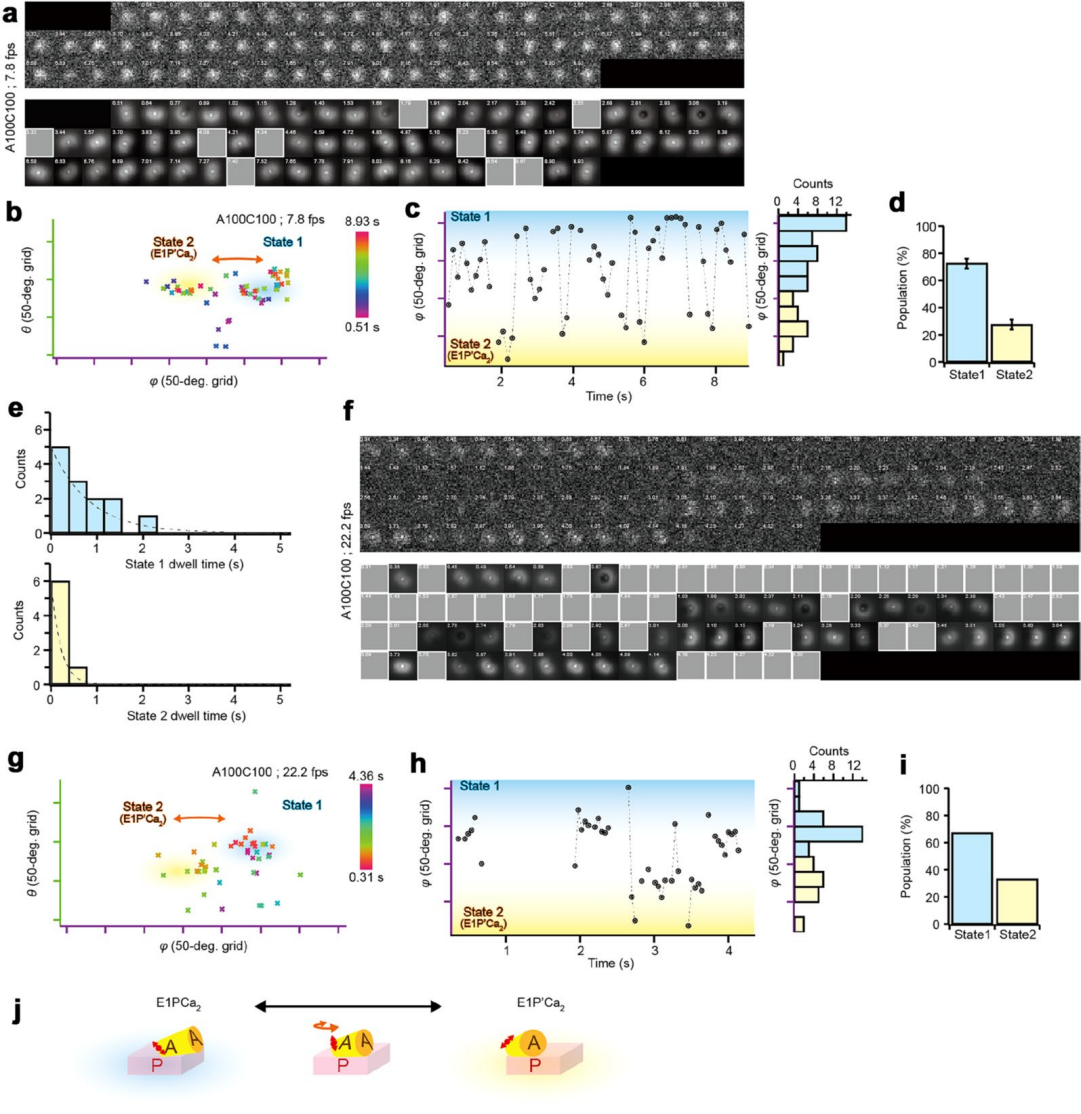
High-speed measurement of the ATP dependent angle change of the SERCA1a A-domain. (**a**) Sequential defocused images of a fluorophore at 100 µM Ca^2+^ and 100 µM ATP (A100C100; cf. Supplementary Video 2). Time scale described in upper left of each image is in seconds. (*Upper panel*) Experimental images were taken at 128 ms intervals. (*Lower panel*) Reconstructed images represent the diffraction pattern rendered using the angles derived from the matching algorithm. Frames covered with gray square are eliminated frames where the matching algorithm failed to estimate angles or estimated incorrect values. (**b**) The *θ* and *φ* angles of the fluorophore at 100 µM Ca^2+^ and 100 µM ATP. The color of each point represents the time, as shown in the color bar. Note that the *θ* and *φ* angles are relative because the orientation of the SERCA1a molecule in a nanodisc is stable but not controlled in our system. (**c**) Time course of the *φ* angle of the fluorophore. (*Right*) Histogram of *φ* angle. Cyan and yellow bars represent States 1 and 2, respectively. (**d**) Populations of States 1 and 2 (*n* = 3 molecules). (**e**) Histograms of the dwell time of State 1 and State 2. (*Upper*) Dwell time of State 1 (*n* = 13 dwells from 3 molecules). Dotted line indicates exponential fits of which the rate constant was ~ 1.2 s^−1^. (*Lower*) Dwell time of State 2 (*n* = 7 dwells from 3 molecules). Dotted line indicates exponential fits of which the rate constant was ~ 4.8 s^−1^. (**f**) Sequential defocused images of a fluorophore at 100 µM Ca^2+^ and 100 µM ATP taken at 45 ms intervals (cf. Supplementary Video 3). (**g**) The *θ* and *φ* angles of the fluorophore. The color of each point represents the time, as shown in the color bar. Note that the *θ* and *φ* angles are relative. (**h**) Time course of the *φ* angle of the fluorophore. (**i**) Populations of States 1 and 2 (*n* = 2 molecules). (**j**) Schematic of the motion of the A-domain, based on the observation in this figure (**a**–**i)** (see “Discussion” section).

To dissect the motion during the transition between State 1 to State 2, we increased the time resolution to 128 ms and 45 ms (Fig. 5a,f and Supplementary Video 2, 3). Note that this high-speed observation was performed within ~ 9 s (128 ms resolution) and ~ 4.5 s (45 ms resolution) because of the photo-bleaching limitation. Under these conditions, the whole ATP-turnover cycle is probably not completed and thus not observable, therefore, we chose only the molecules that showed obvious changes in the angle during observation, and this angle change should occur during the transition between these two states. In the presence of 100 µM ATP and 100 µM Ca^2+^, the trajectory also showed two distinct states (Fig. 5b,g), while the motion was completely absent at low ATP concentration (much lower than *K*_d_ for ATP; 0.001 µM ATP and 1 µM Ca^2+^) or in the presence of TG (Supplementary Fig. S4 and S5). The time course of the *φ* angle revealed back and forth motion between two states (Fig. 5c,h). When the angle changes from State 1 to State 2, the fluorophore stays at State 2 for ~ hundreds of msec, then goes back to State 1. The dwell times of States1 and 2 were fitted by an exponential function of which the rate constants are ~ 1.2 s^−1^ and ~ 4.8 s^−1^ in measurements at 128 ms resolution, respectively (*n* = 13 and 7 dwells from 3 molecules; Fig. 5e). The observed population of State 1 and State 2 are ~ 70% and ~ 30%, respectively, in measurements both at 128 and 45 ms resolution (Fig. 5d,i). These observations suggest the existence of a rapid transition in angle and so a rapid equilibrium of *E*1P and *E*2P-like states (Fig. 5j; see “Discussion” section).

## Discussion

In the last two decades, outstanding studies have revealed the 3D crystal structures of SERCA1a at atomic resolution. The structures of Ca^2+^-transport intermediates provide detailed insights into the conformation of the pump at specific points of the transport cycle. However, the dynamic processes underlying these conformational changes between intermediates are still unknown. Approaches estimating the precise angle of a single fluorescent dipole have enabled us to reveal the true behavior of working proteins in real-time at the single-molecule level^26,31–33^. Even though the spatial resolution has limitation, the time course of the angular changes of a fluorophore, attached to a domain in a single molecule, can reveal an intermediate state^34^. Recently, there have been reports of single-molecule measurements of a P-type ATPase by Förster resonance energy transfer (FRET)^18^ and time-resolved X-ray solution scattering (TR-XSS) combined with Molecular Dynamics simulation^35^. However, our work is the first report of the direct measurement of the angle change of the A-domain in SERCA1a in 3-D space, a critical conformational change in the transport cycle. It provides unprecedented and unequivocal evidence of a swinging A-domain during the structural transition. Further, the spatial and temporal resolution obtained during the dynamics of a working SERCA1a allow measurements of the rates of change and point to an equilibrium of substates in back and forth motion.

In measurements using ligand-stabilized molecules, the observed angle change between the *E*2P analogous state (*E*2·BeF_3_^−^) and the *E*1Ca_2_ state is in excellent agreement with the expected value estimated from crystal structures (Fig. 3e). Therefore, our experimental system is likely capable of measuring the structural changes in other reaction steps of a single SERCA1a molecule. Interestingly, the *θ* angle shows a single population in the *E*2P analogous state (*E*2·BeF_3_^−^) (Fig. 3d). In this state, the cytoplasmic domains of the molecule are tightly gathered into a closed structure^17^. Therefore, if the angle between the fluorophore and the plane parallel to the nanodisc is almost constant, this *θ* angle represents the angle between the glass surface and the nanodisc. Even though the angle of the nanodisc is not controlled because it has only two His-tags, not three points, this single population with a small deviation of *σ*_θ E2BeF_ = 14.3° (Red Bars in Fig. 3d) suggests that the nanodiscs bind to the glass at a particular angle.

In the experiment performed in the presence of Ca^2+^ and ATP, we monitored only the ~ 1% of molecules that showed an obvious change in the angle of the fluorophore. The low percentage can be partially attributed to the selection criteria and to the slow *E*P isomerization compared to the observation time. However, it is possible this proportion may be improved upon. We did test the linker length for the Ni–NTA glass and chose the best linker, C_3_ (~ 1.5 nm; cf. Fig. 2c *inset*), but there may be other approaches. In our conditions, namely in the presence of ATP and an oxygen scavenger, the fluorophore often showed changes in intensity over long periods of time. This may be caused by the motion of the fluorophore, because the intensity of the electromagnetic field generated by the iTIRF depends on the *θ* angle of the fluorophore and on the distance between the glass surface and the fluorophore. A frame of insufficient fluorescence intensity failed to yield estimates or estimated wrong angles from the matching algorithm, and was eliminated (Gray frames of reconstructed images in Figs. 4a,g, 5a,f, Supplementary Fig. S4a,d, S5a,d). This may warrant investigation to improve the proportion of active molecules.

Indeed, the accuracy of the orientation estimation of defocus imaging is reported to be high^19,20^ (accuracy of theta angle estimation was evaluated to ~ 6.3°; see Supplementary Fig. S3), and our measurement system too is highly precise. In the live imaging of the *E*1Ca_2_ state molecule and that stabilized by TG there is a small distribution of *σ*_θ_ = 5.7°–9.2° and *σ*_φ_ = 6.7°–12.7° even under high-speed imaging (Supplementary Fig. S4 and S5). These results indicate ~ 10° precision in our system, as well as showing the molecule is stabilized on the glass. This high accuracy and precision of our system allows for reliable detection of the angle change of the A-domain in *E*P isomerization under live imaging.

In the observation at 1 s resolution (Fig. 4), the trajectory of the angle change in the A-domain is consistent with the direction and track expected from the crystal structures (Supplementary Fig. S1b *Lower*). The observed dwell states, State 1 and State 2, should occur mainly when *E*1PCa_2_ is formed and during *E*2P processing, respectively (Fig. 4j; see “Results” section). The calculated rate constant of the dwell-time in State 2 is ~ 0.2 s^−1^ (Fig. 4f). Considering that State 2 includes transformation of *E*2PCa_2_ to *E*2P and *E*2P hydrolysis, this rate constant is consistent with the rate constant of *E*2P hydrolysis of 0.6–0.3 s^−1^ in the wild type^29,36,37^. The observed angular change between the two dwell states of *ψ*_S1S2_ = 59.5° ± 24.3° (Fig. 4e; see “Results” section), is close to the value expected from the crystal structures, which strongly supports the existence of the dwell states and the pertinence of the rate measurements for the catalytic cycle. Therefore, we can conclude that we succeeded in capturing the motion of a functioning SERCA1a molecule during the reaction cycle, which validates our observation system for measuring domain movements in live imaging.

Under turnover conditions in the observations at both 128 and 45 ms resolution (Fig. 5a–e, f–i, respectively), a frequent change in angle between State 1 and State 2 was detected with an obvious high rate constant of ~ 4.8 s^−1^ from State 2 (Fig. 5e; compare the dwell 2 of *E*2P processing in Fig. 4f and dwell 2 of high-speed observation in Fig. 5e *lower*). This rapid change in angle was never detected at 0.001 µM ATP and 1 µM Ca^2+^, a condition under which the molecules can transform between *E*1Ca_2_ and *E*2 states but cannot form the *E*1PCa_2_ state, and where almost all molecules are in the *E*1Ca_2_ state (Supplementary Fig. S5). Therefore, this rapid change in the angle of the A-domain likely occurs during *E*P isomerization. Since in our experimental condition *E*2P, the ADP-insensitive *E*P, is not accumulated in the steady-state (Fig. 2b *left*), we assigned this State 2 as *E*1P’Ca_2_. This State 2 molecule has an A-domain that is already largely rotated from State 1 (*E*1PCa_2_) to a position close to the *E*2P state, yet seems able to move back rapidly to the State 1 (*E*1PCa_2_) position, indicative of a rapid equilibrium, thus behaving as if it is a transient intermediate *E*2PCa_2_ state (Fig. 5j). This interpretation is consistent with our previous finding in which a proteolytic analysis of *E*2PCa_2_, (captured and stable in the 4Gi-46/47 insertion mutant) indicated that in this state the A-domain is rotated and associated with the P-domain at the tryptic T2 site region (Arg198 on the Val200 loop)^7,8^. Furthermore, the populations of State 1 and State 2, in which the back and forth motion between two states is occurring, suggest *E*1P’Ca_2_ has slightly higher free energy than *E*1PCa_2_. In the observations at both 128 and 45 ms resolution (Fig. 5a–e,f–i, respectively), the ratio of populations of State 2 to State 1 is ~ 0.4 (Fig. 5d,i), which, assuming equilibrium, yields an equilibrium constant of *K*_eq_ ~ 0.4. This value is consistent with the rate constants of dwell-times 1 and 2 (compare the ~ 1.2 s^−1^ in Fig. 5e *upper* and the ~ 4.8 s^−1^ in Fig. 5e *lower*). According to the Gibbs relationship of Δ*G*^0′^ = −*RT*·ln *K*_eq_, the Gibbs free energy of this reaction is only Δ*G*^0′^ ~ 2 kJ/mol.

This observation of frequent back and forth motion and the small difference in free energies suggest that the structural change of the A-domain occurs first, then there is the reduction of Ca^2+^ affinity and gate-opening through the A-domain’s three linkers with large free energy changes, and finally by releasing Ca^2+^ the protein is transformed to the *E*2P ground state, abbreviated as *E*2PCa_2_[occluded] → *E*2PCa_2_(lumenally open high-affinity) → *E*2PCa_2_(lumenally open low-affinity) → *E*2P + 2Ca^2+^, see Ref.^38^. Thus, our live imaging of a single membrane molecule at work is important not only for further understanding of the Ca^2+^ transport mechanism of SERCA but also for exploring the properties of other P-type ATPases.

## Methods

### Mutagenesis and expression

The pMT2 expression vector^39^ carrying rabbit SERCA1a cDNA^40^ with TC motif (Cys–Cys–Pro–Gly–Cys–Cys) inserted between Asp196 and Pro197 in the A-domain (TCi-196/197 mutant) was constructed as described previously^8^. Transfection of pMT2 DNA into COS-1 cells and preparation of microsomes from the cells were performed as described^41^. The monkey cell line derived from kidney, COS-1 (RCB0143), was provided by the RIKEN BRC through the National Bio-Resource Project of the MEXT/AMED, Japan.

### ReAsH-labeling of SERCA1a microsomes and preparation of nanodisc containing a single labeled Ca^2+^-ATPase

The TCi-196/197 or wild type SERCA1a was labeled with ReAsH reagent essentially according to the method of Chen et al.^42^ used for FlAsH labeling. The TCi-196/197 or wild type SERCA1a microsomes (2.4 mg mL^−1^) prepared from COS-1 cells were pretreated with 10 mM β-mercaptoethanol and 10 mM tris (carboxyethyl)phosphine in 150 mM MOPS/Tris (pH 7.0) for 1 h at 25 °C. Then 15 μM ReAsH-EDT_2_ was added to the medium and incubated for a further 30 min. To stop the binding reaction, the samples were diluted threefold into 150 mM MOPS/Tris (pH 7.0) containing 50 μM 2,3-dimercapto-1-propanesulfonic acid (stop solution). The unbound probe was separated from microsomes by centrifugation (100,000×*g* for 10 min).

For measurement of the total amount of *E*P at steady-state and the *E*2P fraction, the resulting pellet was resuspended in 0.1 mM CaCl_2_, 0.1 M KCl, 0.3 M sucrose, and 5 mM MOPS/Tris (pH 7.0). Aliquots of the reaction were taken for determination of *E*P formation at 0 and 30 min. For the zero point, the stop solution was added immediately before the addition of ReAsH solution.

The labeled SERCA1a protein was inserted into nanodiscs using the following procedure. The ReAsH-labeled microsomes (0.224 mg mL^−1^) were incubated with 0.5 mg mL^−1^ native sarcoplasmic reticulum vesicles, 20 μM MSP1D1 (membrane scaffold protein 1D1) in 1.1 mM 1-palmitoyl-2-oleoyl-sn-glycero-3-phosphocholine (POPC), 10 mM CaCl_2_, 20 mM Tris/HCl (pH 7.5), and 10 mg mL^−1^ octaethylene glycol monododecyl ether (C_12_E_8_) on ice for 30 min. Then the nanodiscs containing a ReAsH labeled or a native SERCA1a were reconstituted, and this mixture was purified by size exclusion column chromatography as described previously^24^. Both the ReAsH-labeled SERCA1a and the native SERCA1a embedded nanodiscs were infused into the flow chamber for single-molecule imaging, and fluorescence from only the ReAsH-labeled molecules was observed.

### Ca^2+^-ATPase activity

Ca^2+^-ATPase activity of expressed SERCA1a was obtained essentially as described previously^43^. The rate of ATP hydrolysis was determined at 25 °C in a mixture containing 1 μg of microsomal protein, 0.1 mM [γ-^32^P]ATP, 0.1 M KCl, 7 mM MgCl_2_, 10 μM CaCl_2_, 1 μM A23187, and 50 mM MOPS/Tris (pH 7.0). The Ca^2+^-ATPase activity of expressed SERCA1a was obtained by subtracting the ATPase activity determined in the presence of 1 μM TG, a highly specific and subnanomolar affinity SERCA inhibitor with conditions otherwise as above.

### Total amount of EP (total EP, sum of E1P and E2P) at steady state and E2P fraction

Phosphorylation of SERCA1a in microsomes with [γ-^32^P]ATP was performed essentially as described previously^37^. Microsomes expressing wild type or TCi-196/197 mutant SERCA1a were phosphorylated with [γ-^32^P]ATP at 0 °C for 30 s in 50 μl of a mixture containing 1.2 μg of microsomal protein, 10 μM [γ-^32^P]ATP, 1 μM A23187, 0.1 M KCl, 7 mM MgCl_2_, 10 μM CaCl_2_, and 50 mM MOPS/Tris (pH 7.0). The total *E*P formed was determined after acid quenching. For determination of ADP-insensitive *E*P (*E*2P), an equal volume of a mixture containing 2 mM ADP, 1 μM A23187, 0.1 M KCl, 7 mM MgCl_2_, 10 mM EGTA, and 50 mM MOPS/Tris (pH 7.0) was added to the above phosphorylation mixture, and the reaction was quenched at 1 s after the ADP addition. ADP-sensitive *E*P (*E*1P) disappears entirely within 1 s after the ADP addition.

Precipitated proteins were separated by 5% SDS-PAGE at pH 6.0 according to Weber and Osborn^44^. The radioactivity associated with the separated Ca^2+^-ATPase was quantified by digital autoradiography as described^45^. The amount of *E*P in expressed SERCA1a was obtained by subtracting the background radioactivity determined in the presence of 1 μM TG, with conditions otherwise as above. We confirmed that 1 μM TG reduces the *E*P value in the wild type and all mutants to a background radioactivity level (i.e., 1% of the maximum *E*P level, which is the same as that obtained in the absence of Ca^2+^ without TG).

### Observation of a single ReAsH-attached SERCA1a molecule

Flow chambers were made with a width of 3 mm on Ni–NTA coated glass (32 × 24 mm; NEO, Matsunami Glass Industry), and an untreated cover glass (1818 No.1; Matsunami Glass Industry) was placed on top. The Ni–NTA coated glass was made using a protocol described previously^46^. We infused 3 chamber volumes (CV; ~ 8 µL) of observation-buffer (50 mM MOPS/Tris pH 7.0, 0.1 M KCl, and 7 mM MgCl_2_) containing 100 µM CaCl_2_, and irradiated with an excitation laser for 13.5 min to reduce the background signal. Then, we infused 3 CVs of observation-buffer containing 100 µM CaCl_2_ and 3 mg mL^−1^ BSA (centrifuged then passed through a *φ* = 0.22 µm pore filter), and irradiated with an excitation laser for 13.5 min. To attach the SERCA1a embedded nanodisc, 1 CV of ~ 15 µg mL^–1^ molecules in observation-buffer containing 100 µM CaCl_2_ was infused. After incubation for 5 min, 6 CVs of A0.001C1-buffer (observation-buffer containing 0.001 µM ATP, and 1 µM CaCl_2_), A0.01C1-buffer (observation-buffer containing 0.01 µM ATP, and 1 µM CaCl_2_), A1C100-buffer (observation-buffer containing 1 µM ATP, and 100 µM CaCl_2_), and A100C100-buffer (observation-buffer containing 100 µM ATP, and 100 µM CaCl_2_) containing the ATP-regenerating system (0.020 mg mL^−1^ creatine kinase and 0.082 mg mL^−1^ creatine phosphate)^34,47^ with the oxygen scavenger system (1% β-mercaptoethanol, 4.5 mg mL^−1^ glucose, 0.25 mg mL^−1^ glucose oxidase, and 90 U mL^−1^ catalase)^21,48^ were infused.

### Observation of the ligand-stabilized SERCA1a

*E*2·BeF_3_^−^ formation was induced by a method based on that previously described^49^. The SERCA1a embedded nanodiscs (~ 15 µg mL^−1^) were incubated for 45 min at 23 °C in BeF_3_^–^ buffer (50 mM MOPS/Tris at pH 7.0, 50 mM LiCl, 5 mM MgCl_2_, 10 µM CaCl_2_, 0.5 mM KF, and 40 µM BeSO_4_). The *E*2P analogous state (*E*2·BeF_3_^−^) SERCA1a was observed in the BeF_3_^–^ buffer containing the oxygen scavenger system.

*E*1Ca_2_ formation was induced by the following method: The SERCA1a embedded nanodiscs were incubated for 5 min at 23 °C in observation-buffer containing 20 mM CaCl_2_ and the oxygen scavenger system.

*E*2(TG) formation was induced by the following method: The SERCA1a embedded nanodiscs were incubated for 5 min at 23 °C (for Fig. 3f,g) or at 5 °C (for Supplementary Fig. S4) in observation-buffer containing 1 µM CaCl_2_, 100 µM ATP, 3 µM thapsigargin, and the oxygen scavenger system.

### Microscopy and defocused imaging

A ReAsH-attached SERCA1a was visualized under an inverted microscope (TE2000E; Nikon Instruments) equipped with a 100 × objective lens (Apo TIRF 1.49 N.A.; Nikon Instruments), a 532-nm laser (JUNO532-800; Showa Optronics) with custom-made dichroic mirror to keep the laser polarization after reflection (Chroma), two emission filters (NF03-532E & NF01-532U; Semrock), an EMCCD camera (iXon^+^ DU897; Andor), a highly stable customized stage (Chukosha), and an optical table (Newport). The detailed optical setup was described previously^21,25^. All systems were set into a compartment (Nihon Freezer) under which the temperature was stabilized at 23.0 ± 0.1 °C or 5.0 ± 0.1 °C by PID regulation with a heater and a cooler.

The isotropic TIRFM was constructed using a diffractive diffuser (D0740A, Thorlabs) based on the method reported previously^21,25^. To observe defocused images, the distance between the objective lens and the specimen was kept constant by the perfect focus system (Nikon Instruments)^21^. The custom-made piezoelectric stage (P-620.ZCL; Physik Instrumente GmbH & Co) was used for calibration of the exact distance^50^.

In the observation performed at 5 °C, to decrease the aberration, the objective lens was typically placed ~ 640 nm closer beyond the best focal plane, and the correction collar was set at the position fully turned counterclockwise.

### Analyzing the defocused image

Each defocused image was analyzed using 3D steerable filters, based on the method reported previously^20,21^. To solve the equation for estimation of dipole orientation (see Appendix A1 in Ref^20^), we used the custom software reported in the Ref^21^. Parameters for determining the optical path difference^51^ used the following values: Refractive index of glass is 1.526; Refractive index of immersion is 1.518; Thickness of glass is measured by the micrometer (MDC-25MX, Mitutoyo; typically 0.144 mm); Design value for thickness of glass is the value of the objective correction collar (typically 0.150 mm). Note that we take into consideration the effect of the glass equipped in the iXon EMCCD camera.

All *θ* and *φ* angles were validated by comparing the reconstructed images and experimental images. The defocused image of the fluorophore has inherent degeneracy due to fluorophore symmetry along its dipole axis, i.e., (*θ*, *φ*) is equivalent to (180° − *θ*, *φ *− 180°). Therefore, in live imaging (Figs. 4, 5), we selected the angle either (*θ*, *φ*)_next_ or (180° − *θ*, *φ *− 180°)_next_, which had a smaller change from (*θ*_prev_, *φ*_prev_) in the previous frame.

Evaluation of the theta angle estimation was performed by the following method. The diffraction patterns were rendered by the method reported previously^20^. Poisson noise was generated using RandomJ plugin for Image J and added to the simulated patterns. RMSE (root mean square error) was calculated by Igor Pro (WaveMetrics). Orientation estimation of *θ* angle was performed under the condition in which the *φ* value was fixed at 0°.

### Comparison of crystal structures

The angle between *E*2P (*E*2·BeF_3_^−^) and *E*1Ca_2_ was calculated 56.4°, 51.0°, 53.0°, and 54.8° from structures of 3B9B^2^ for *E*2·BeF_3_^−^ and 1SU4^6^, 2C9M^52^, 3J7T^53^, and 5XA7^54^ for *E*1Ca_2_, respectively. Note that 1SU4^6^, 2C9M^52^, 3J7T^53^, and 5XA7^54^ were categorized *E*1Ca_2_ state in Ref^55^. The angle between *E*1PCa_2_ (*E*1Ca_2_·AlF_4_^−^·ADP) and *E*2P (*E*2·BeF_3_^−^) was calculated 58.8° from structures: (PDB ID code: 1T5T^56^ and 2ZBE^17^). We aligned two molecules using M7-M10 helices with the measure fit command included in VMD software^57^. The *ψ* angle was defined as the angle derived from the inner product of each unit vector along residues 196 to 197 on the A-domain.

### Statistics and reproducibility

The number of individual experiments is in the corresponding figure legend. In particular, all molecules of Figs. 3, 4, 5 and Supplementary Fig. S4, S5 were obtained from four replicate assays in each condition. All molecules were selected according to the criteria described in the main text and representative molecules are shown. In the control experiments of Fig. 4g–i and Supplementary Figs. S4, S5, we analyzed ~ 4000 bright spots in each condition and verified that all bright spots did not show a change in angles. All error bars shown are the standard deviation.

## Supplementary information

Supplementary Video 1.

Supplementary Video 2.

Supplementary Video 3.

Supplementary Informations.

## Data Availability

The data that support the findings of this study are available from the corresponding authors upon reasonable request.

## References

[1] Toyoshima C, Nomura H, Tsuda T (2004). Lumenal gating mechanism revealed in calcium pump crystal structures with phosphate analogues. Nature.

[2] Olesen C (2007). The structural basis of calcium transport by the calcium pump. Nature.

[3] Møller JV, Juul B, Maire MI (1996). Structural organization, ion transport, and energy transduction of P-type ATPases. Biochim. Biophys. Acta.

[4] Moller JV, Olesen C, Winther AM, Nissen P (2010). The sarcoplasmic Ca2+-ATPase: Design of a perfect chemi-osmotic pump. Q. Rev. Biophys..

[5] Ebashi S, Lipmann F (1962). Adenosine triphosphate-linked concentration of calcium ions in a particulate fraction of rabbit muscle. J. Cell. Biol..

[6] Toyoshima C, Nakasako M, Nomura H, Ogawa H (2000). Crystal structure of the calcium pump of sarcoplasmic reticulum at 2.6 A resolution. Nature.

[7] Daiho T, Danko S, Yamasaki K, Suzuki H (2010). Stable structural analog of Ca2+-ATPase ADP-insensitive phosphoenzyme with occluded Ca2+ formed by elongation of A-domain/M1'-linker and beryllium fluoride binding. J. Biol. Chem..

[8] Daiho T, Yamasaki K, Danko S, Suzuki H (2007). Critical role of Glu40-Ser48 loop linking actuator domain and first transmembrane helix of Ca2+-ATPase in Ca2+ deocclusion and release from ADP-insensitive phosphoenzyme. J. Biol. Chem..

[9] Daiho T, Yamasaki K, Danko S, Suzuki H (2014). Second transmembrane helix (M2) and long range coupling in Ca(2)(+)-ATPase. J. Biol. Chem..

[10] Moller JV (2002). Calcium transport by sarcoplasmic reticulum Ca(2+)-ATPase. Role of the A domain and its C-terminal link with the transmembrane region. J. Biol. Chem..

[11] Lenoir G (2004). Functional properties of sarcoplasmic reticulum Ca(2+)-ATPase after proteolytic cleavage at Leu119-Lys120, close to the A-domain. J. Biol. Chem..

[12] Holdensen AN, Andersen JP (2009). The length of the A-M3 linker is a crucial determinant of the rate of the Ca2+ transport cycle of sarcoplasmic reticulum Ca2+-ATPase. J. Biol. Chem..

[13] Daiho T (2003). Deletions of any single residues in Glu40-Ser48 loop connecting a domain and the first transmembrane helix of sarcoplasmic reticulum Ca(2+)-ATPase result in almost complete inhibition of conformational transition and hydrolysis of phosphoenzyme intermediate. J. Biol. Chem..

[14] Danko S, Yamasaki K, Daiho T, Suzuki H, Toyoshima C (2001). Organization of cytoplasmic domains of sarcoplasmic reticulum Ca(2+)-ATPase in E(1)P and E(1)ATP states: A limited proteolysis study. FEBS Lett..

[15] Toyoshima C (2013). Crystal structures of the calcium pump and sarcolipin in the Mg2+-bound E1 state. Nature.

[16] Tsunekawa N, Ogawa H, Tsueda J, Akiba T, Toyoshima C (2018). Mechanism of the E2 to E1 transition in Ca(2+) pump revealed by crystal structures of gating residue mutants. Proc. Natl. Acad. Sci. U. S. A..

[17] Toyoshima C, Norimatsu Y, Iwasawa S, Tsuda T, Ogawa H (2007). How processing of aspartylphosphate is coupled to lumenal gating of the ion pathway in the calcium pump. Proc. Natl. Acad. Sci. U. S. A..

[18] Dyla M (2017). Dynamics of P-type ATPase transport revealed by single-molecule FRET. Nature.

[19] Toprak E (2006). Defocused orientation and position imaging (DOPI) of myosin V. Proc. Natl. Acad. Sci. U. S. A..

[20] Aguet F, Geissbühler S, Märki I, Lasser T, Unser M (2009). Super-resolution orientation estimation and localization of fluorescent dipoles using 3-D steerable filters. Opt. Express.

[21] Fujimura S (2017). Dissection of the angle of single fluorophore attached to the nucleotide in corkscrewing microtubules. Biochem. Biophys. Res. Commun..

[22] Danko S, Yamasaki K, Daiho T, Suzuki H (2004). Distinct natures of beryllium fluoride-bound, aluminum fluoride-bound, and magnesium fluoride-bound stable analogues of an ADP-insensitive phosphoenzyme intermediate of sarcoplasmic reticulum Ca2+-ATPase: Changes in catalytic and transport sites during phosphoenzyme hydrolysis. J. Biol. Chem..

[23] Griffin BA, Adams SR, Tsien RY (1998). Specific covalent labeling of recombinant protein molecules inside live cells. Science.

[24] Yamasaki K, Daiho T, Danko S, Yasuda S, Suzuki H (2017). Nanodisc-based kinetic assays reveal distinct effects of phospholipid headgroups on the phosphoenzyme transition of sarcoplasmic reticulum Ca(2+)-ATPase. J. Biol. Chem..

[25] Nishizaka T, Hasimoto Y, Masaike T (2011). Simultaneous observation of chemomechanical coupling of a molecular motor. Methods Mol. Biol..

[26] Forkey JN, Quinlan ME, Shaw MA, Corrie JET, Goldman YE (2003). Three-dimensional structural dynamics of myosin V by single-molecule fluorescence polarization. Nature.

[27] Sagara Y, Inesi G (1991). Inhibition of the sarcoplasmic reticulum Ca2+ transport ATPase by thapsigargin at subnanomolar concentrations. J. Biol. Chem..

[28] Toyoshima C, Nomura H (2002). Structural changes in the calcium pump accompanying the dissociation of calcium. Nature.

[29] Yamasaki K, Daiho T, Danko S, Suzuki H (2004). Multiple and distinct effects of mutations of Tyr122, Glu123, Arg324, and Arg334 involved in interactions between the top part of second and fourth transmembrane helices in sarcoplasmic reticulum Ca2+-ATPase: Changes in cytoplasmic domain organization during isometric transition of phosphoenzyme intermediate and subsequent Ca2+ release. J. Biol. Chem..

[30] Daiho T, Kubota T, Kanazawa T (1993). Stoichiometry of tight binding of magnesium and fluoride to phosphorylation and high-affinity binding of ATP, vanadate, and calcium in the sarcoplasmic reticulum calcium-ATPase. Biochemistry.

[31] Ha T, Enderle T, Chemla DS, Selvin PR, Weiss S (1996). Single molecule dynamics studied by polarization modulation. Phys. Rev. Lett..

[32] Nishizaka T (2004). Chemomechanical coupling in F1-ATPase revealed by simultaneous observation of nucleotide kinetics and rotation. Nat. Struct. Mol. Biol..

[33] Forkey JN, Quinlan ME, Goldman YE (2005). Measurement of single macromolecule orientation by total internal reflection fluorescence polarization microscopy. Biophys J.

[34] Masaike T, Koyama-Horibe F, Oiwa K, Yoshida M, Nishizaka T (2008). Cooperative three-step motions in catalytic subunits of F(1)-ATPase correlate with 80 degrees and 40 degrees substep rotations. Nat. Struct. Mol. Biol..

[35] Ravishankar H (2020). Tracking Ca(2+) ATPase intermediates in real time by x-ray solution scattering. Sci. Adv..

[36] Wang G, Yamasaki K, Daiho T, Suzuki H (2005). Critical hydrophobic interactions between phosphorylation and actuator domains of Ca2+-ATPase for hydrolysis of phosphorylated intermediate. J. Biol. Chem..

[37] Daiho T, Yamasaki K, Danko S, Suzuki H (2016). Glycine 105 as pivot for a critical knee-like joint between cytoplasmic and transmembrane segments of the second transmembrane helix in Ca2+-ATPase. J. Biol. Chem..

[38] Danko S, Yamasaki K, Daiho T, Suzuki H (2017). Membrane Perturbation of ADP-insensitive phosphoenzyme of Ca(2+)-ATPase modifies gathering of transmembrane helix M2 with cytoplasmic domains and luminal gating. Sci. Rep..

[39] Kaufman RJ, Davies MV, Pathak VK, Hershey JW (1989). The phosphorylation state of eucaryotic initiation factor 2 alters translational efficiency of specific mRNAs. Mol. Cell. Biol..

[40] Brandl CJ, Green NM, Korczak B, MacLennan DH (1986). Two Ca2+ ATPase genes: Homologies and mechanistic implications of deduced amino acid sequences. Cell.

[41] Maruyama K, MacLennan DH (1988). Mutation of aspartic acid-351, lysine-352, and lysine-515 alters the Ca2+ transport activity of the Ca2+-ATPase expressed in COS-1 cells. Proc. Natl. Acad. Sci. U.S.A..

[42] Chen B, Mahaney JE, Mayer MU, Bigelow DJ, Squier TC (2008). Concerted but noncooperative activation of nucleotide and actuator domains of the Ca-ATPase upon calcium binding. Biochemistry.

[43] Daiho T (2001). Mutations of either or both Cys876 and Cys888 residues of sarcoplasmic reticulum Ca2+-ATPase result in a complete loss of Ca2+ transport activity without a loss of Ca2+-dependent ATPase activity. Role of the CYS876-CYS888 disulfide bond. J. Biol. Chem..

[44] Weber K, Osborn M (1969). The reliability of molecular weight determinations by dodecyl sulfate-polyacrylamide gel electrophoresis. J. Biol. Chem..

[45] Daiho T, Suzuki H, Yamasaki K, Saino T, Kanazawa T (1999). Mutations of Arg198 in sarcoplasmic reticulum Ca2+-ATPase cause inhibition of hydrolysis of the phosphoenzyme intermediate formed from inorganic phosphate. FEBS Lett..

[46] Naito TM (2019). Single-molecule pull-out manipulation of the shaft of the rotary motor F1-ATPase. Sci. Rep..

[47] Katoh TA (2018). Three-dimensional tracking of microbeads attached to the tip of single isolated tracheal cilia beating under external load. Sci. Rep..

[48] Harada Y, Sakurada K, Aoki T, Thomas DD, Yanagida T (1990). Mechanochemical coupling in actomyosin energy transduction studied by in vitro movement assay. J. Mol. Biol..

[49] Danko S, Daiho T, Yamasaki K, Liu X, Suzuki H (2009). Formation of the stable structural analog of ADP-sensitive phosphoenzyme of Ca2+-ATPase with occluded Ca2+ by beryllium fluoride: Structural changes during phosphorylation and isomerization. J. Biol. Chem..

[50] Katoh, T. A., Fujimura, S. & Nishizaka, T. In *Handbook of Photonics for Biomedical Engineering* (eds A. H.-P. Ho, D. Kim, & M. G. Somekh) 755–766 (Springer, 2017).

[51] Gibson SF, Lanni F (1991). Experimental test of an analytical model of aberration in an oil-immersion objective lens used in three-dimensional light microscopy. J. Opt. Soc. Am. A.

[52] Jensen AM, Sorensen TL, Olesen C, Moller JV, Nissen P (2006). Modulatory and catalytic modes of ATP binding by the calcium pump. EMBO J..

[53] Yonekura K, Kato K, Ogasawara M, Tomita M, Toyoshima C (2015). Electron crystallography of ultrathin 3D protein crystals: Atomic model with charges. Proc. Natl. Acad. Sci. U S A.

[54] Norimatsu Y, Hasegawa K, Shimizu N, Toyoshima C (2017). Protein-phospholipid interplay revealed with crystals of a calcium pump. Nature.

[55] Aguayo-Ortiz R, Espinoza-Fonseca LM (2020). Linking biochemical and structural states of SERCA: Achievements, challenges, and new opportunities. Int. J. Mol. Sci..

[56] Sørensen TL-M, Møller JV, Nissen P (2004). Phosphoryl transfer and calcium ion occlusion in the calcium pump. Science.

[57] Humphrey W, Dalke A, Schulten K (1996). VMD: Visual molecular dynamics. J. Mol. Graph..

