# Supplementary material for: Angle change of the A-domain in a single SERCA1a molecule detected by defocused orientation imaging
Source: Sci Rep. 2021 Jul 1;11:13672. doi: 10.1038/s41598-021-92986-3 (PMC8249593; doi:10.1038/s41598-021-92986-3)
Supplement: Supplementary file 4 — Supplementary Informations. [file 41598_2021_92986_MOESM4_ESM.docx]

**Supplementary Information**

Angle change of the A-domain in a single SERCA1a molecule detected by defocused orientation imaging

Takanobu A. Katoh^1,#^, Takashi Daiho^2^, Kazuo Yamasaki^2^, Stefania Danko^2^, Shoko Fujimura^1,§^, Hiroshi Suzuki^2^

^1^Department of Physics, Faculty of Science, Gakushuin University, Toshima-ku Tokyo 171-8588, Japan. ^2^Department of Biochemistry, Asahikawa Medical University, Midorigaoka-higashi, Asahikawa 078-8510, Japan

Present address: ^#^Laboratory for Organismal Patterning, RIKEN Center for Biosystems Dynamics Research, RIKEN, Minatojima-minamimachi, Chuo-ku, Kobe, Hyogo 650-0047, Japan. ^§^AIST-UTokyo Advanced Operando-Measurement Technology Open Innovation Laboratory (OPERANDO-OIL), National Institute of Advanced Industrial Science and Technology (AIST), Kashiwa, 277-8565, Japan.

Correspondence should be addressed to T.A.K. (takanobu.a.katoh@gmail.com) and H.S. (hisuzuki@asahikawa-med.ac.jp).


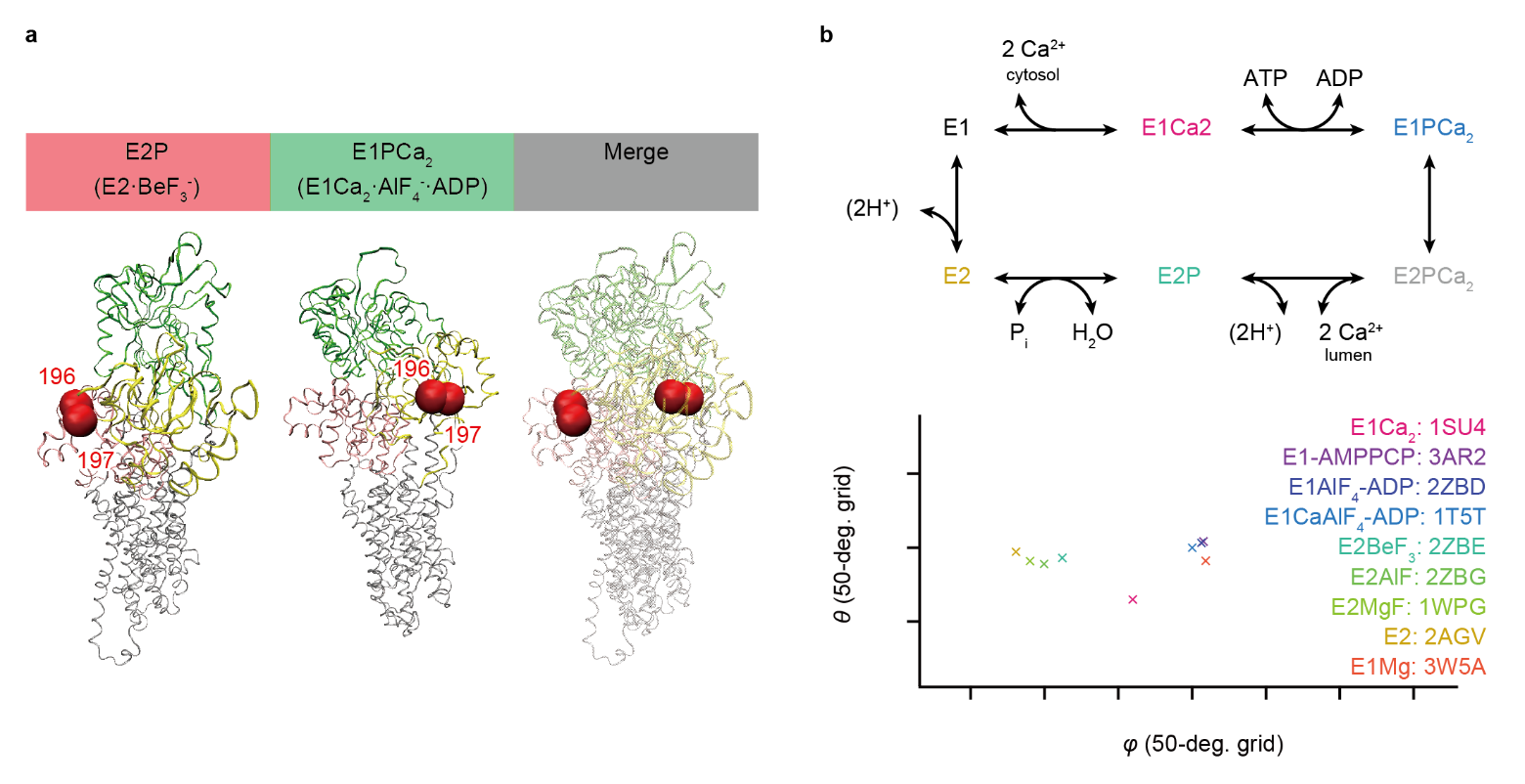


**Supplementary Fig. S1: Location of the tetra-cysteine insertion and the zenith and azimuth angles derived from crystal structures.**

**(a)** Residues Asp196 and Pro197 in A-domain (Red spheres). These residues are postulated to display a large change in angle between the *E*1PCa_2_ and the *E*2P models (*E*1Ca_2_·AlF_4_^-^·ADP and *E*2·BeF_3_^-^ crystal; PDB ID code: 1T5T and 2ZBE). Tetra-cysteine (TC) was inserted between these residues. A fluorophore, ReAsH, was attached to the A-domain at fixed orientation via TC. The crystal structures in the *E*2P (*E*2·BeF_3_^-^) and *E*1PCa_2_ states (PDB ID code: 3B9B and 1SU4; Two molecules are aligned using M7-M10 helices). **(b)** Reaction scheme of the Ca^2+^-ATPase (*Upper*), and the expected *θ* and *φ* angles between residues Asp196 and Pro197 in crystal structures (*Lower*). We defined the origin of the angles as the *θ* and *φ* angles in the *E*1Ca_2_·AlF_4_^-^·ADP crystal structure (*E*1PCa_2_ model, PDB ID code: 1T5T; see Methods).

**
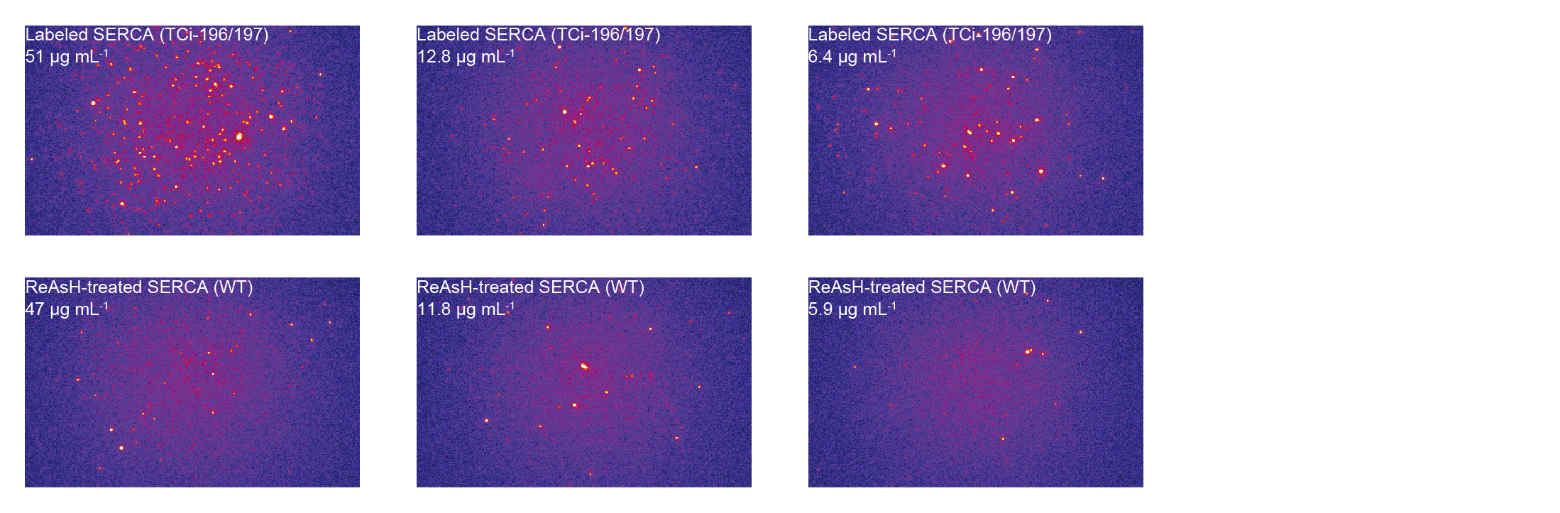
Supplementary Fig. S2: Fluorescence images of ReAsH-bound and -unbound SERCA1a containing nanodiscs fixed on Ni-NTA coated glass via His-tags.**

Fluorescent images of fluorophores attached to the SERCA1a (TCi-196/197; *Upper*) and that of the ReAsH-treated non-TC-tagged SERCA1a (WT; *Lower*). Red dots represent fluorophores. While the number of bright spots in labeled SERCA1a depends on the concentration, it is almost constant in Non-labeled SERCA1a (See Fig. 2d).


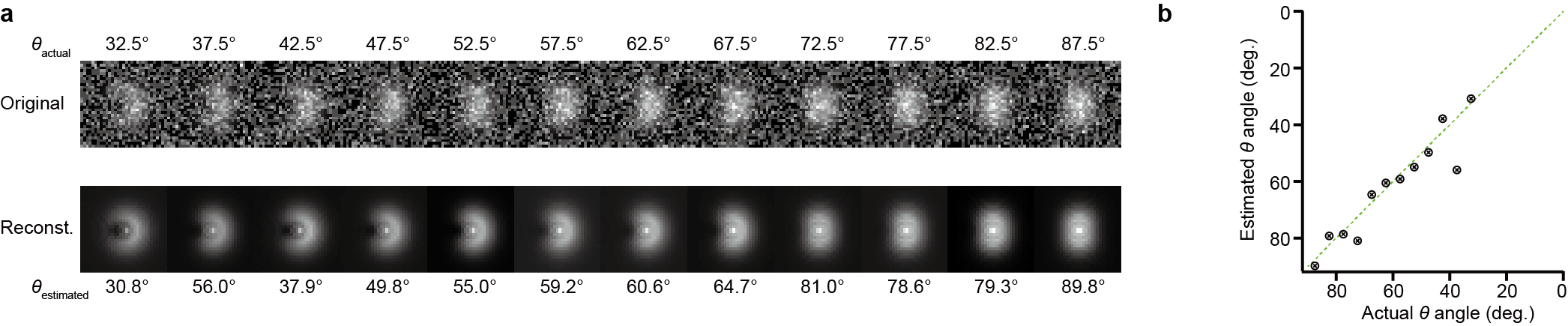


**Supplementary Fig. S3: Evaluation of the theta angle estimation on simulated data.**

**(a)** Simulated diffraction patterns rendered by the formation model of defocused imaging (*Upper panel*). All images had Poisson noise added. Actual theta angle was written as *θ*_actual_. Reconstructed images using the angles derived from the matching algorithm (*Lower panel*). Estimated theta angle was written as *θ*_estimated_. **(b)** Evaluation of the theta angle estimation. Black markers represent the estimated *θ* angle from the noisy original images displayed in upper panel of (a). Green dotted line indicates actual angle. The RMSE (root mean square error) was calculated to 6.3°.

**
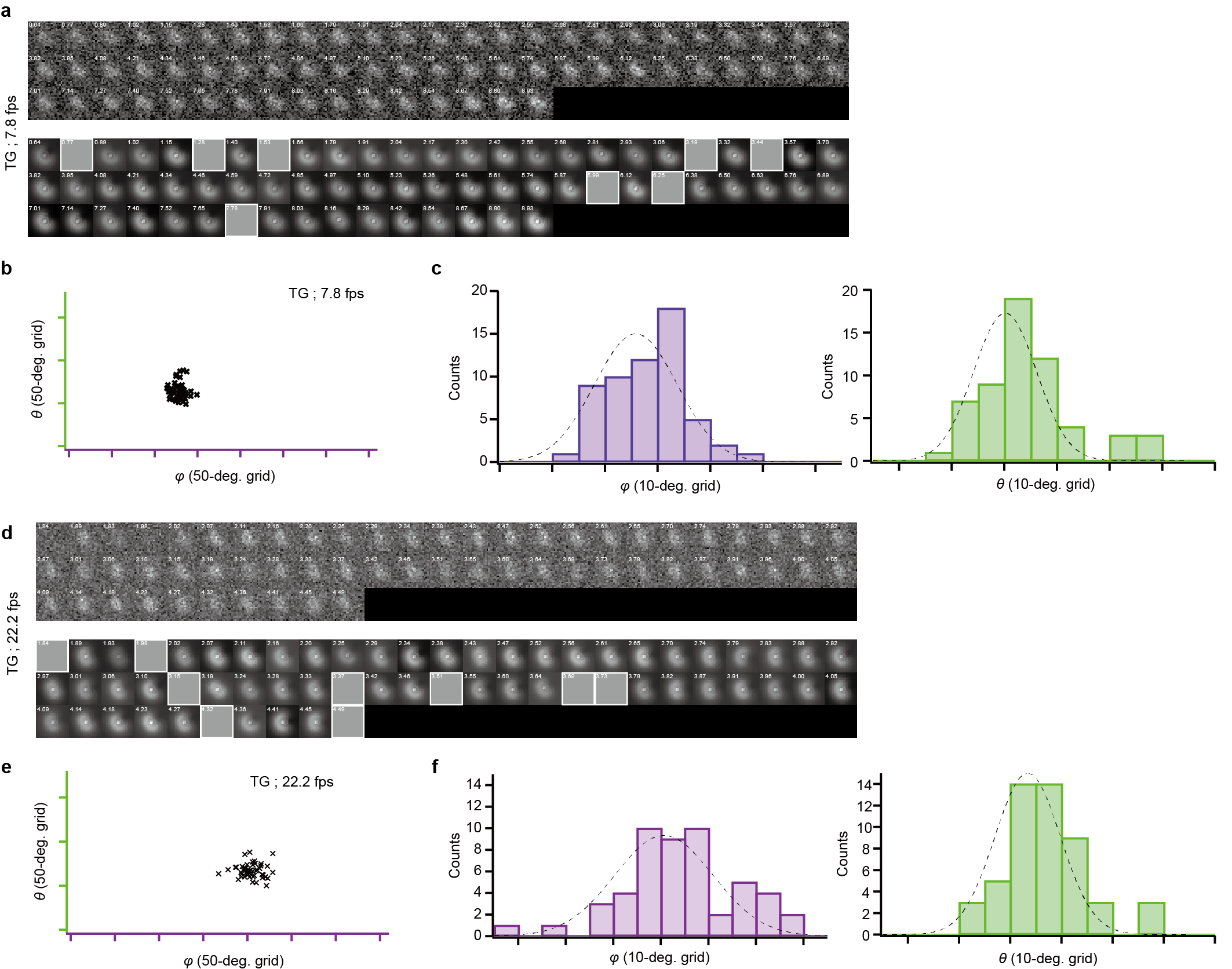
**

**Supplementary Fig. S4: *θ* and *φ* angles of A-domain in SERCA1a stabilized by TG.**

**(a)** Sequential defocused images of a fluorophore. Time scale described in upper left of each image is in seconds. (*Upper panel*) Experimental images were taken at 128 ms intervals. No molecules showed a change in angles. (*Lower panel*) Reconstructed images using the angles derived from the matching algorithm. Frames covered with gray square are eliminated frames where the matching algorithm failed to estimate angles or estimated incorrect values. **(b)** The *θ* and *φ* angles of the fluorophore in (a). **(c)** (*left*) A histogram of the *φ* angle. The dotted line indicates the Gaussian fit, where *σ*_φ_ = 7.9°. (*right*) A histogram of the *θ* angle. The dotted line indicates the Gaussian fit, where *σ*_θ_ = 6.0°. **(d)** Sequential defocused images of a fluorophore. Images were taken at 45 ms intervals. No molecules showed a change in angles. **(e)** The *θ* and *φ* angles of the fluorophore in (d). **(f)** (*left*) A histogram of the *φ* angle. The dotted line indicates Gaussian fit, where *σ*_φ_ = 10.3°. (*right*) A histogram of the *θ* angle. The dotted line indicates Gaussian fit, where *σ*_θ_ = 6.4°.


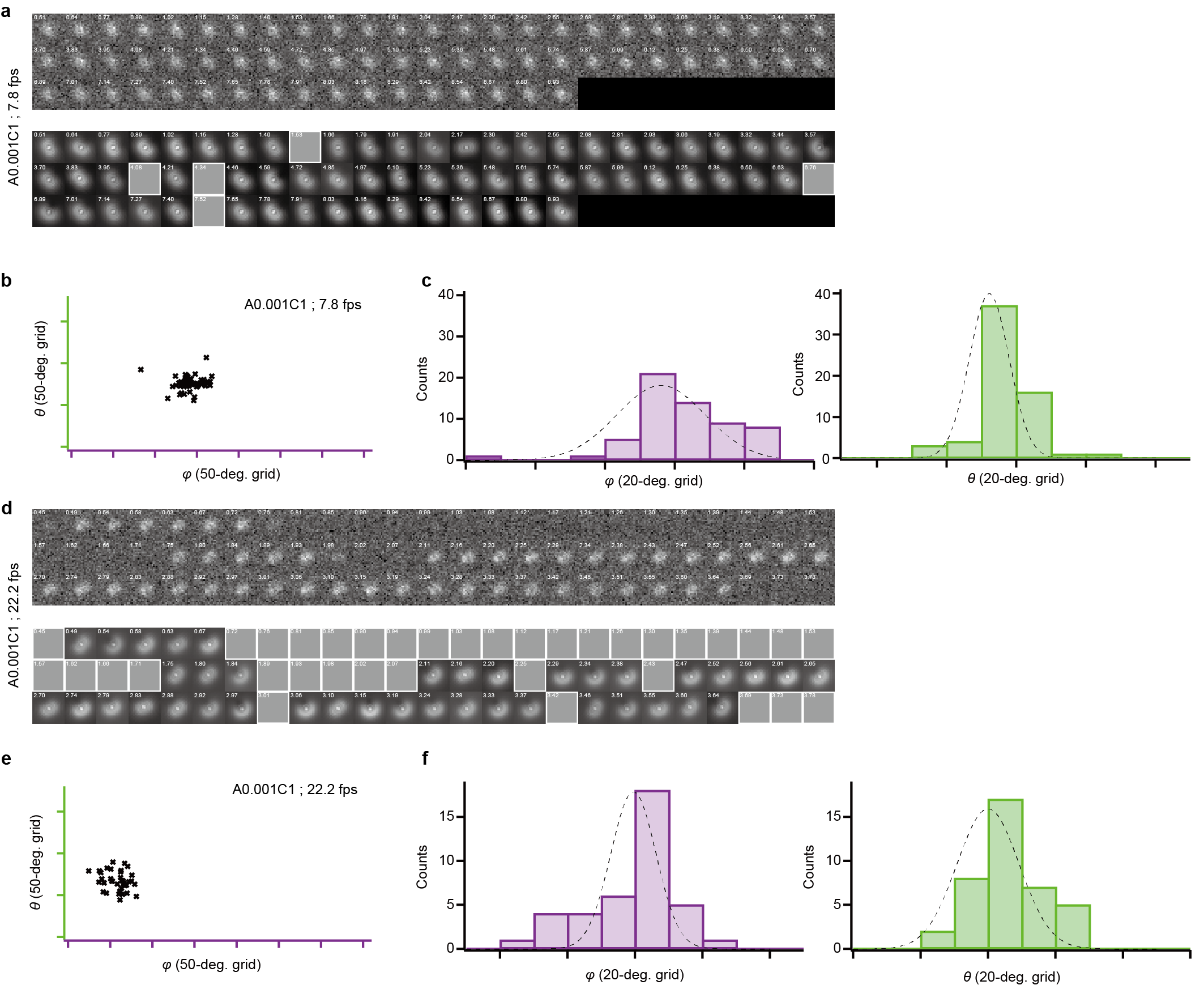


**Supplementary Fig. S5: Measurement of the angles of A-domain in SERCA1a at subsaturating ATP concentration**

**(a)** Sequential defocused images of a fluorophore at 1 µM Ca^2+^ and 0.001 µM ATP (A0.001C1). In this condition, almost all molecules form the *E*1Ca_2_ state, yet they can transform between *E*1Ca_2_ and *E*2 states. Time scale described in upper left of each image is in seconds. (*Upper panel*) Experimental images taken at 128 ms intervals. No molecules showed a change in angles. (*Lower panel*) Reconstructed images using the angles derived from the matching algorithm. Frames covered with gray square are eliminated frames where the matching algorithm failed to estimate angles or estimated incorrect values. **(b)** The *θ* and *φ* angles of the fluorophore in (a). **(c)** (*left*) A histogram of the *φ* angle. The dotted line indicates the Gaussian fit, where *σ*_φ_ = 12.7°. (*right*) A histogram of the *θ* angle. The dotted line indicates the Gaussian fit, where *σ*_θ_ = 5.7°. **(d)** Sequential defocused images of a fluorophore. Images were taken at 45 ms intervals. No molecules showed a change in angles. **(e)** The *θ* and *φ* angles of the fluorophore in (d). **(f)** (*left*) A histogram of the *φ* angle. The dotted line indicates Gaussian fit, where *σ*_φ_ = 6.7°. (*right*) A histogram of the *θ* angle. The dotted line indicates Gaussian fit, where *σ*_θ_ = 9.2°.

**Description of Additional Supplementary Files**

**File Name: Supplementary Video 1**

**Description:** The time-course of the change in angle of the A-domain at 1 µM ATP and 100 µM Ca^2+^ with a time resolution of 1 s. The upper left, upper right, and lower panel show the defocused images of a fluorophore, reconstructed images, and estimated *θ* and *φ* angles of the fluorophore, respectively (see Fig. 4a,c).

**File Name: Supplementary Video 2**

**Description:** The time-course of the change in angle of the A-domain at 100 µM ATP and 100 µM Ca^2+^ with a time resolution of 128 ms. The upper left, upper right, and lower panel show the defocused images of a fluorophore, reconstructed images, and estimated *θ* and *φ* angles of the fluorophore, respectively (see Fig. 5a,b).

**File Name: Supplementary Video 3**

**Description:** The time-course of the change in angle of the A-domain at 100 µM ATP and 100 µM Ca^2+^ with a time resolution of 45 ms. The upper left, upper right, and lower panel show the defocused images of a fluorophore, reconstructed images, and estimated *θ* and *φ* angles of the fluorophore, respectively (see Fig. 5f,g).
